# Train related injuries: A descriptive analysis highlighting orthopaedic injuries and management

**DOI:** 10.1051/sicotj/2021038

**Published:** 2021-08-17

**Authors:** Christina Niovi Kontoghiorghe, Simon Matthew Graham, Joel Rodriguez, Richard Matzopoulos, Sithombo Maqungo

**Affiliations:** 1 Division of Orthopaedic Surgery, Groote Schuur Hospital 7935 Cape Town South Africa; 2 University College London Hospitals NHS Foundation Trust NW1 2BU London UK; 3 Institute of Population Health Sciences, University of Liverpool L69 3BX Liverpool UK; 4 Department of Orthopaedic and Trauma Surgery, Liverpool University Teaching Hospital Trust L9 7AL Liverpool UK; 5 University of Texas Southwestern Medical Center Dallas 75390 TX USA; 6 Burden of Disease Research Unit, South African Medical Research Council 7505 Cape Town South Africa; 7 School of Public Health and Family Medicine, University of Cape Town 7925 Cape Town South Africa; 8 Division of Global Surgery, University of Cape Town 7925 Cape Town South Africa

**Keywords:** Train accidents, Orthopaedic injuries, Amputation, Limb salvage

## Abstract

*Introduction*: Orthopaedic injuries constitute a major aspect of morbidity and mortality following train accidents. The pattern of orthopaedic/musculoskeletal injuries sustained following these accidents has not been fully characterised. The main aim of this study is to describe the range of orthopaedic injuries reported in a major trauma centre and evaluate their management, as well as reporting mortality and amputation rates. Further aims are to identify the social and demographic background of the patients to suggest treatment and prevention strategies. *Methods*: This study is a retrospective observation of all clinical files of patients presented to Level 1 Trauma Centre in Cape Town, South Africa, as “train casualty” from January 2013 to July 2019, which were reviewed and evaluated. A total of 174 patients were included, of which 92 were orthopaedic referrals. The average age was 29 years, and 87% were male. *Results*: Tibial fractures were most common (*N* = 19), 38% of patients sustained open fractures, and 68% of patients (in total) underwent surgery. Wound debridement was the most common operation, followed by open reduction internal fixation (ORIF). Twelve patients (13%) underwent amputation to 14 body parts. Eight patients (4.6%) (in total) died in the trauma unit. *Discussion*: This study provides insight into train accident victims and their orthopaedic injuries and management patterns. The victims are largely young males. The majority of orthopaedic injuries require surgical intervention, and those who make it to the hospital have a good chance of survival and limb salvage. It appears that in addition to early hospital access and specialised updated treatments, morbidity and mortality in train accidents could be reduced by improving safety measures and social awareness to reduce railway violence and accidents.

## Introduction

Railway transportation is typically one of the safest modes of transport, however, railway accidents occur throughout the world [1]. In 2018–2019, South Africa, a country with a population of around 57 million, saw 174.6 million passenger journeys with 641 fatalities and weighted injuries on their railway network [2, 3]. This number is high compared to other places such as the UK (population over 66 million), which saw 318 fatalities in 2019–2020 with 1742.4 million passenger journeys [4, 5].

In South Africa, trains are often overcrowded and pass through underdeveloped urban areas to carry commuters to their place of work in the city centre. In Cape Town, one of the major cities of South Africa with a population surpassing 3.7 million people, up to a quarter of journeys are made using public transport [6, 7]. The city’s railway network comprises of 118 stations, and there are an estimated 621,833 journeys a day [8].

A wide spectrum of injuries results from train accidents, and with already limited resources, this places an additional burden on the healthcare system of South Africa. The majority of the population are reliant on public healthcare, which is already weighed down by the high prevalence of HIV/tuberculosis, maternal, and child mortality, as well as injury and trauma resulting from high crime rates [9].

We noted many patients presenting to our hospital – a Level 1 Trauma Centre in Cape Town, South Africa, following train accidents. Matzopoulos and Lerer have extensively researched railway injuries in Cape Town, but no focused analysis of orthopaedic injuries and management has been made [10–12]. Singer et al. reported orthopaedic injuries from train accidents in Cape Town over thirty years ago [13]. Singer and Donnally (USA) have more recently described their findings of orthopaedic injuries related to train accidents [14, 15]. The detail on the pattern of orthopaedic injuries and their management from a lower-income country is lacking or out of date. This study sets out to describe this and determine if there has been any progress over the years.

The primary aim of this study is to describe the range of orthopaedic injuries reported as a result of train accidents and evaluate their management, as well as to report mortality and amputation rates. Further aims are to describe the social and demographic details of all patients presenting from a train injury to our hospital’s trauma unit to raise awareness and help design treatment and prevention strategies for reducing related morbidity and mortality.

## Material and methods

This is a retrospective observational study of all patients and their injuries presented to the Groote Schuur Hospital (GSH) – a Level 1 Trauma Centre in Cape Town, South Africa, involved in train accidents from January 2013 to July 2019.

Inclusion criteria for the study were all patients presenting with reference “train casualty” to the trauma unit. Exclusion criteria for the study were any patient without a medical file; any patient for whom the mechanism of injury did not fit the criteria of a train accident when reviewed in the notes – including an injury sustained at the train station but not related to being on or near a train; any patient presenting later than the date of injury, and also any patient with insufficient documentation in their files.

The registers from the trauma unit were reviewed retrospectively from prospectively entered data. One individual collected data on a set proforma to ensure consistency and the patients’ medical files were obtained from the medical records department. Demographic and clinical data were collected and uploaded onto a Microsoft Excel (Microsoft Corporation, 2018) spreadsheet for analysis.

**Figure 1:**
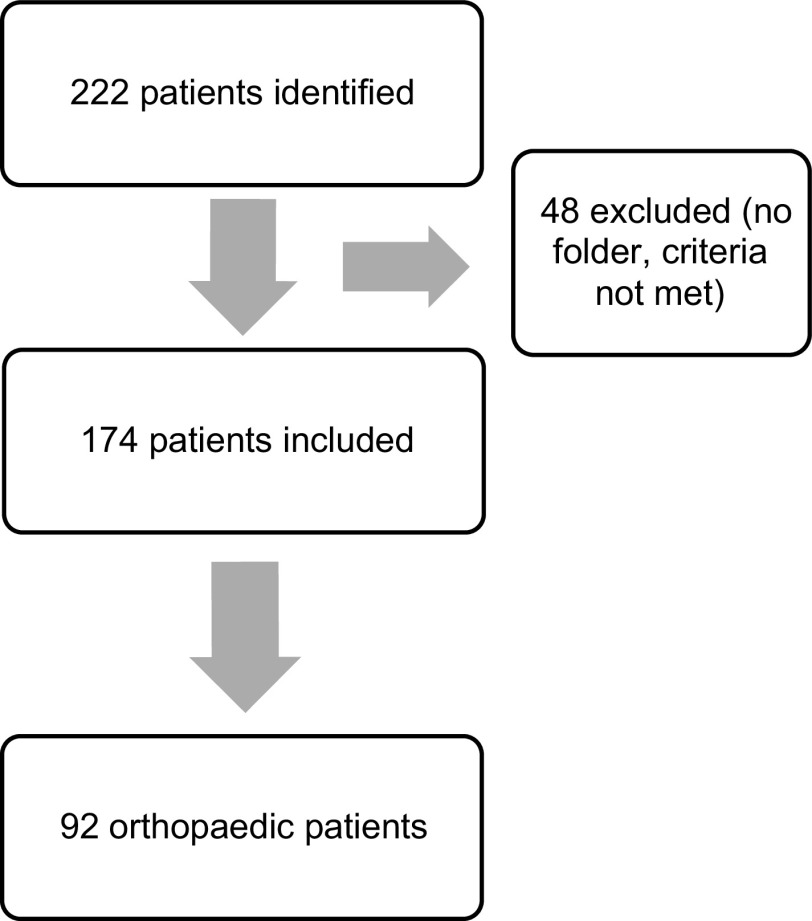
Patients included in the study. Two-hundred and twenty-two patients initially identified, 48 excluded; a total of 174 patients were included in the study and 92 of these were referred to the orthopaedic unit.

A total of 51 months of admissions to the trauma unit spanning seven years were analysed. Three registers were missing from July 2013 to April 2014, January 2015 to November 2015, and April 2018 to September 2018. Two hundred and twenty-two patients were identified as presenting as train casualties to the GSH trauma department. Forty-eight patients were excluded, leaving a total of 174 patients included in the study; 92 of these sustained musculoskeletal injuries and were referred to the orthopaedic department (Figure 1). All patients were admitted under ATLS (Advanced Trauma Life Support) certified specialists in the trauma unit.

As this is a descriptive analysis of our findings, there was no null hypothesis and no power calculations were carried out. Categorical data are presented in frequencies and percentages; continuous data are reported as mean and standard deviation. Age is reported as median and interquartile range (IQR).

All data were collected in line with the hospital and university data collection policies. All patient-identifiable data were omitted. Ethical approval from both GSH and the University of Cape Town Human Research Ethics Committee was sought and granted (DRC REF 2019/068, HREC REF: 419/2019).

## Results

### Orthopaedic patients

Of the 174 patients included in the study, 92 (53%) were referred to the orthopaedic department with musculoskeletal injuries. A total of 61 out of 92 patients had surgery for orthopaedic injuries. There were 145 theatre encounters for 172 procedures (some patients underwent multiple procedures under the same anaesthetic). Table 1 shows the orthopaedic procedures and complications. Males accounted for 89% of patients, and the average age was 31 years (range 12–83; IQR 14).

**Table 1:** Orthopaedic operations and complications.

Operation	Number (% of operations)
Intramedullary nailing	10 (5.8)
Open reduction internal fixation (plate)	35 (20.3)
External fixation	11 (6.4)
Ring fixation	1 (0.6)
Removal of ex-fix/wires	9 (5.2)
Removal of metalwork	8 (4.7)
Skin graft	8 (4.7)
Skin flap	2 (1.2)
Wound debridement	61 (35.5)
K-wire fixation	3 (1.7)
Manipulation under anaesthesia	3 (1.7)
Ligament reconstruction	1 (0.6)
Limb amputation	20 (11.6)
Upper limb	1 (0.6)
Lower limb	19 (11.0)
(Foot)	2 (1.2)
(Below knee)	7 (4.1)
(Through knee)	5 (2.9)
(Above knee)	5 (2.9)
Total operations	172
Total theatre encounters	145
Complications	
Wound sepsis	4 (40.0)
Non-union	1 (10.0)
Failure of fixation	1 (10.0)
Nerve injury	1 (10.0)
Infected metalwork	1 (10.0)
Post-operative stiffness	1 (10.0)
Heterotopic ossification	1 (10.0)
Total complications	10

A total of 172 operations were reported. The commonest operation was wound debridements (35.5% of operations), followed by ORIF (20.3%), and 20 amputation procedures. Ten post-operative complications were reported in total, the majority of which were wound infections (40%).

**Figure 2:**
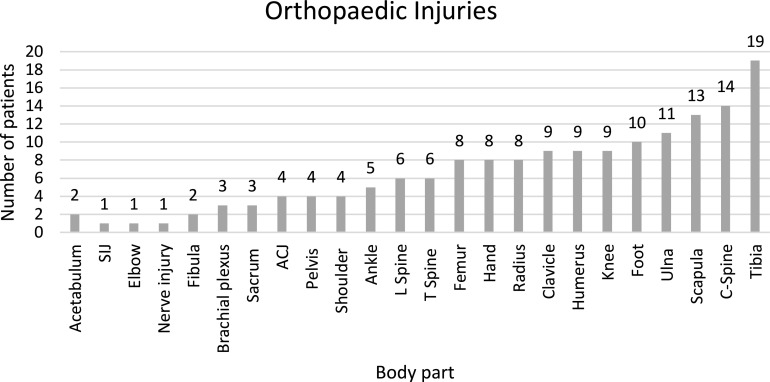
Orthopaedic injuries. Presentation of data showing the frequency of injuries incurred by body part. The most common fracture was the tibia (*N* = 19), followed by C-spine (*N* = 14).

Each orthopaedic patient had, on average musculoskeletal injuries to 1.74 body parts (range 1–6; SD 1.04). Figure 2 shows the orthopaedic injuries sustained. The injuries incurred included fractures and nerve injuries. Thirty-five patients sustained open fractures (38%). Non-operative management was undertaken in 32% of patients. Operative management took place in 68% of patients. Nineteen patients who underwent surgery also had concomitant fractures managed conservatively. Conservative management mainly was for spinal fractures (Philadelphia collar, cones calipers, thoracic lumbar sacral orthosis (TLSO) bracing), clavicle/scapula/shoulder injuries (collar and cuff). Undisplaced fractures of the ankle or forearm (including distal radius) were usually treated with plaster casting.

In total, 12 patients (6.9%) underwent amputations to 14 body parts. Ten of these patients had suffered traumatic amputations on arrival to 12 body parts. Two patients underwent amputation for unsalvageable limb injuries; one open comminuted patella fracture required an above knee amputation (AKA), and one mangled foot (Mangled Extremity Severity Score (MESS) = 7) required a below knee amputation (BKA). The majority of patients did not have a documented mechanism of injury, but for those available, two had been pushed, one had jumped, and one had been train surfing.

The majority of patients were haemodynamically stable on presentation (86%). Eleven patients required blood transfusion with an average of 2 units (range 1–4). Revised Trauma Score (RTS) was available for 65 patients, mean 7.5 (range 3–8; SD 1.05). Eighty-six percent of patients (79/92) required outpatient follow-up. Table 2 shows the comparison of injuries in all patients versus orthopaedic patients.

**Table 2:** Comparison of injuries in all patients versus orthopaedic patients.

	All patients *N* (%)	Orthopaedic patients *N* (%)
Injury		
Head	88 (50.3)	38 (41.3)
Face	35 (20.2)	16 (17.4)
Neck	1 (0.6)	1 (1.1)
Thorax	35 (20.2)	19 (20.7)
Abdomen	23 (13.3)	12 (13.0)
Pelvis	5 (2.9)	5 (5.4)
Spine	27 (15.6)	25 (27.2)
Upper limb	50 (28.9)	45 (48.9)
Lower limb	54 (31.2)	45 (48.9)
Vascular	1 (0.6)	1 (1.1)

Head injuries occurred in 50.3% of all patients. Of the orthopaedic patients, upper and lower limb injuries occur simultaneously (48.9%). Spinal injuries occurred in 27.2% of orthopaedic patients.

### Summary of all patients

Seventy-four percent (128/174) of patients were admitted, and 3.4% (6/174) died in the trauma unit. Two further patients died shortly after admission, bringing the total number of deaths to 8 (4.7%) (Table 3).

**Table 3:** Comparison of all patients versus orthopaedic patient demographics.

	All patients *N* (%)	Orthopaedic patients *N* (%)
Sex		
Male	152 (87.4)	82 (89.1)
Female	22 (12.6)	10 (10.9)
Median age (years)	29 (IQR 13.0)	31 (IQR 14)
Employment		
Employed	43 (24.7)	–
Unemployed	71 (40.8)	–
Student	9 (5.2)	–
Disabled	1 (0.6)	–
Pensioner	2 (1.1)	–
Unknown	48 (27.6)	–
Mechanism		
Jumping on/off train	15 (8.6)	12 (13.0)
Fell	38 (21.8)	14 (15.2)
Injured on train	1 (0.6)	0 (0.0)
Crossing railway line	8 (4.6)	5 (5.4)
Suicide	0 (0.0)	0 (0.0)
Pushed	52 (29.9)	27 (29.3)
Train surfing	5 (2.9)	3 (3.3)
Unknown	34 (19.5)	21 (22.8)
Other	21 (12.1)	10 (10.9)
Outcome		
Admitted	128 (73.6)	82 (89.1)
Discharged	38 (21.8)	9 (9.9)
Died	6 (3.4)	1 (1.1)
Unknown	2 (1.1)	0 (0.0)
ICU	15 (8.8)	8 (8.9)
Total deaths	8 (4.6)	2 (2.2)

Most victims are unemployed young males. The majority are injured, pushed, or falling from the train. About 8% of patients required intensive care and less than 5% total fatalities.

Of the eight patients who died from their injuries, seven were male, and the average age was 36 years (range 24–77; IQR 28.3). Only one patient was haemodynamically stable on presentation to the trauma department. Seven patients had head injuries. There was one facial injury, three thoracic injuries, one abdominal injury, three spinal, one upper limb, and one lower limb injury. RTS was available for five patients and the mean score was 5.8 (range 4–7; SD 1.1). Two patients were admitted to the intensive care unit (ICU) and the length of stay was four and six days. Although one presented in cardiac arrest, three patients suffered orthopaedic injuries, so was not referred to the orthopaedic team. This patient had fractures in the right femur and right humerus. The other two patients had C-spine fractures, namely: C5–C6 facet fractures left transverse process fracture C7, and fracture of the C5 spinous process. The other patient suffered C5–6 bi-facet dislocation.

Eighty-seven percent (152/174) of all train injury patients were male. The average age was 29 years (range 12–83; IQR 13.0). Table 3 shows the demographics of these patients, including the mechanism of injury. Sixty-five percent (113/174) of victims came from the Klipfontein (old Cape Flats) neighbourhood in Cape Town, with 16% (28/174) from the Northern suburbs, and 10% (17/174) from the Southern Suburbs.

**Figure 3:**
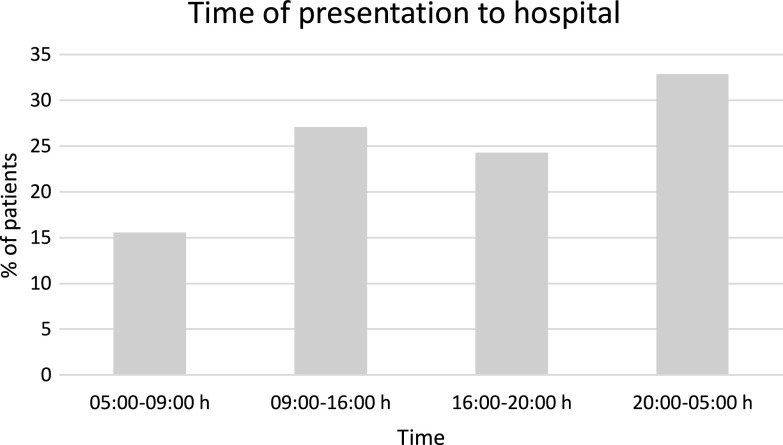
Time of presentation to hospital. Presentation of data showing the percentage (%) number of patients presenting during each time period throughout the day. Most patients presented during the night to early morning (20:00–05:00 h) 32.9%, fewest patients during morning commute 15.6%.

Figure 3 shows the time of presentation to the hospital, with most patients arriving between 20:00 h and 05:00 h. The number of cases varies throughout the year. January saw the fewest cases (7/174) compared to May, which had the most number cases (22/174).

## Discussion

This study describes the orthopaedic injuries and management as well as demographics of train accident victims presented to Level 1 Trauma Centre in Cape Town, South Africa, over 7 years. The study found that most orthopaedic patients required surgical intervention and that amputation rates as well as mortality rates were low.

Over two-thirds of orthopaedic patients required surgery. Those undergoing surgery underwent, on average 2.38 procedures. Overall, 12 patients underwent 20 amputation procedures to 14 body parts, only one of these was the upper extremity. The most commonly performed operations were debridement and open reduction internal fixation (ORIF). The debridement of wounds occurred mainly for open fractures and amputation stumps before definitive closure. The rate of postoperative complications was low and was determined by the available post-operative and outpatient follow up documentation. The average length of hospital stay for orthopaedic patients was 14.9 days, and 87% had outpatient follow up.

The study demonstrates a high burden of orthopaedic trauma as a result of train accidents at our hospital. The patients admitted to hospital required high levels of intervention through multidisciplinary reviews and surgical procedures and lengthy inpatient stays. This increases pressure on the department with regard to theatre time and inpatient capacity. Nevertheless, the low death rate (4.7%) and low complication rate for patients admitted under the orthopaedic team are encouraging and suggest that these patients’ management and their injuries are appropriate and effective. Just under 7% of patients underwent amputations, and this figure is far lower than the rate of fixation for fractures (>47% of patients).

Patients sustaining orthopaedic (musculoskeletal) injuries have similar characteristics to the entire cohort. They also suffer high rates of concurrent head, thoracic, and abdominal trauma. Their lengthy inpatient stay and high follow up rate implies the complexity of their injuries. Often patients required multiple returns to theatre, usually for soft tissue debridement in the case of amputations or open fractures. This supports the notion that limb salvage does have a place over amputation in this patient cohort, particularly when considering the psychological and economic consequences in the future.

The low rate of amputation found in this study (6.9%) is comparable with the results from a previous study by Singer et al. performed at the same hospital in 1988, where 6.2% of patients required amputation [13]. This number remains low and may indicate that amputation was and still is being carried out as a last resort for unsalvageable limbs.

The majority of train injury victims were young males. A large number were not formally employed. The overall unemployment rate in Cape Town in 2020 was 24.8%; the percentage of patients unemployed in our study, 40.8%, is higher than this average [16]. Nearly all patients came from poor socioeconomic areas.

**Figure 4:**
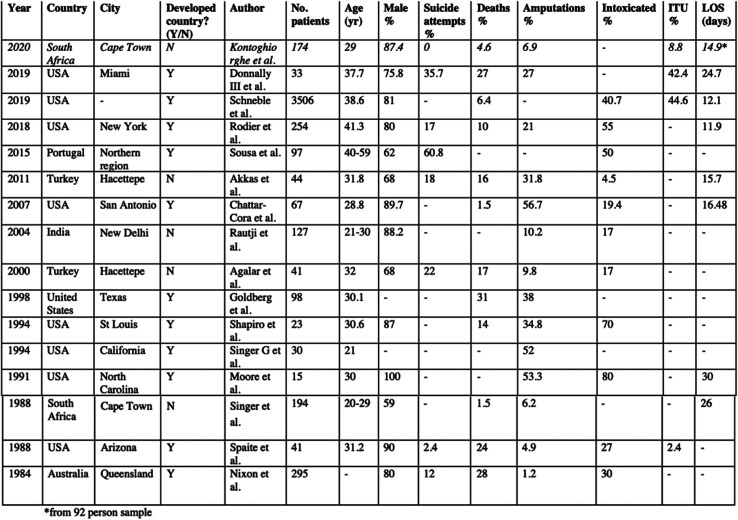
Summary of literature review findings. Salient points are noted, including the year of publication, country/city, whether this is a developed or developing country, authors, number of patients in the study, mean age, percentage male, suicide attempts, mortality rate, amputation rate, intoxication rate, percentage of patients requiring intensive care admission, and the average length of stay (days).

The study findings keep with what we know from previous reports that victims of train accidents are mostly male and in their late 20s or early 30s (Figure 4) [13–15, 17–28]. The rates of suicide attempts in this study and generally in the developing world are much lower than the models demonstrated in developed countries [24, 29]. In comparison to studies on railway accidents undertaken in Cape Town over 30 years ago, there is a much higher proportion of injuries resulting from violence in this study [13]. The percentage of injuries occurring during the morning and evening commutes are comparable, but the highest number of injuries occur during the evening to night-time hours. There already exists a spread of fatality rates as a result of these accidents in the literature, however, a number of deaths in our study is on the lower side, as is the rate of amputation. Although we have not investigated the correlation of alcohol with our patients involved in train accidents, several previous studies have found a high prevalence of intoxication amongst train accident victims (Figure 4).

There are a number of limitations to this study. The presented data were subject to human error when entered and transferred in the notes and is especially reliant on emergency service and nursing notes. Analysis was made of available data and may not be accurate when missing data are taken into account. The employment status is self-reported and may not be accurate. This is a retrospective study and, therefore a lower level of evidence. The study was carried out at a single trauma centre in one city in South Africa and may not represent the wider region or country. Only victims who survived the initial trauma and were brought to hospital are featured in the study. Therefore the number of fatalities from train accidents is likely to be much higher than the number who died after presentation to the trauma unit. Children are underrepresented in the study as in most circumstances are not admitted to GSH. No information was available for analysis regarding alcohol or drug intoxication. The severity of the injuries and their impact on patient outcomes is not evaluated in detail.

In the USA, there were higher rates of open fractures and suicide as well as preference for primary amputation as opposed to limb salvage [15, 19]. In New Delhi, India, high rates of trespassing are a leading cause of railway fatalities. In Turkey there were both higher amputation and higher death rates, with increased rates of suicide attempts and cases of persons hit by the train [25, 27].

Paniker et al. outlined how the global burden of road trauma could be reduced, and railroad trauma is no different [30]. Increased awareness through publicity is key, and this has been shown by Sousa et al., where fatality rates dropped following a television campaign [24]. National and international organisations and governments must recognise the high morbidity and mortality associated with these injuries and direct funding and research towards developing and implementing safer practices on the railways.

## Conclusion

The findings of this study provide insight into train accident victims and their orthopaedic injuries and management with large sample size. The victims who make it to the hospital have a good chance of survival and even limb salvage. However, tighter controls of railway violence and increased health and safety measures must be implemented to minimise injuries and reduce mortality on the railways in Cape Town. Campaigns targeting high-risk groups – young males from poor socioeconomic areas, could raise awareness about railway safety and reduce the burden of train accidents.

Future studies where possible should be designed in a prospective manner incorporating train accident victims from throughout the region or country with coding of injuries and operations to ensure validity and reliability. It would be of economic importance to calculate the financial burden of these injuries and how they affect return to employment, as well as evaluating the proportion of victims who are intoxicated to focus preventative approaches and strategies even further.

## Conflict of interest

The authors declare that they have no relevant financial or non-financial interests to report.

## Funding

This research did not receive any specific funding.

## Ethical approval

This study received ethical approval from the Ethics committee of Groote Schuur Hospital and University of Cape Town under the protocol number DRC REF 2019/068, HREC REF: 419/2019.

## Informed consent

Not deemed applicable in this retrospective observational study.

## Author contributions

Christina Niovi Kontoghiorghe – study design, data collection, writing, editing, submitting; Simon Matthew Graham – reviewing and editing; Joel Rodriguez – study design, data collection; Richard Matzopoulos – reviewing and editing; Sithombo Maqungo – conceptualisation, methodology, supervision, reviewing and editing.

## References

[1] International Railway Safety Council (2021) Safety stastics. In: Railw. Saf., International Rail Safety Council. https://international-railway-safety-council.com/safety-statistics/.

[2] Railway Safety Regulator State of safety report 2018/2019.

[3] Department: Statistics South Africa (2020) STATISTICAL RELEASE P7162 Land transport (Preliminary) May 2020.

[4] Office of Rail and Road (2020) Rail safety. https://dataportal.orr.gov.uk/statistics/health-and-safety/rail-safety/. Accessed 28 Dec 2020.

[5] Office of Rail and Road (2020) Passenger rail usage. In: Table 1220 – Passeng. journeys. https://dataportal.orr.gov.uk/statistics/usage/passenger-rail-usage/table-1220-passenger-journeys/. Accessed 12 Apr 2021.

[6] Department of statistics South Africa (2011) Statistics by place. http://www.statssa.gov.za/?page_id=964. Accessed 3 Feb 2021.

[7] Deloitte City Mobility Index Cape Town. https://www2.deloitte.com/content/dam/insights/us/articles/4331_Deloitte-City-Mobility-Index/CapeTown_GlobalCityMobility_WEB.pdf. Accessed 23 Jul 2020.

[8] RAIL NETWORK. https://www.tct.gov.za/en/transport/transport-network/rail-network/. Accessed 23 Jul 2020.

[9] World Health Organization (2018) South Africa. In: Ctry. Coop. Strateg. a glance. file:///Users/cnk/Downloads/ccsbrief_zaf_en.pdf. Accessed 3 Feb 2021.

[10] Lerer LB, Matzopoulos RG (1997) Fatal railway injuries in Cape Town, South Africa. Am J Forensic Med Pathol. 10.1097/00000433-199706000-00007. 9185930

[11] Lerer LB, Matzopoulos R (1996) Meeting the challenge of railway injury in a South African city. Lancet. 10.1016/S0140-6736(96)02100-9. 8782759

[12] Matzopoulos R, Lerer LB (1998) Hours to hell and back: A social epidemiology of railway injury in a South African city, 1890–1995. Soc Sci Med. 10.1016/S0277-9536(98)00043-4. 9683381

[13] Singer M, Anderson PPA (1988) Train accidents and orthopaedic morbidity. The Groote Schuur Hospital experience. South African Med J. 3340929

[14] Singer G, Thordarson D (1994) Train-versus-pedestrian injuries. Orthopaedic management. Orthop Rev. 8196964

[15] Donnally CJ, Sheu JI, Roth ES, et al. (2019) An elevated metrorail as a source of orthopedic injuries and death at a Level-I Trauma Center. Iowa Orthop J. PMC6604534 31413689

[16] Democratic Alliance (2020) Cape Town has lowest unemployment rate of all metros. https://www.da.org.za/government/where-we-govern/2020/02/cape-town-has-lowest-unemployment-rate-of-all-metros. Accessed 15 Aug 2020.

[17] Goldberg BA, Mootha RK, Lindsey RW (1998) Train accidents involving pedestrians, motor vehicles, and motorcycles. Am J Orthop. 9586732

[18] Shapiro MJ, Luchtefeld WB, Durham RM, Mazuski JE (1994) Traumatic train injuries. Am J Emerg Med. 10.1016/0735-6757(94)90210-0. 8285985

[19] Moore TJ, Wilson JR, Hartman M (1991) Train versus pedestrian accidents. South Med J. 10.1097/00007611-199109000-00009. 1891729

[20] Spaite D, Criss E, Valenzuela T, et al. (1988) Railroad accidents: A metropolitan experience of death and injury. Ann Emerg Med. 10.1016/S0196-0644(88)80404-9. 3377292

[21] Nixon J, Corcoran A, Fielding L, Eastgate J (1985) Fatal and nonfatal accidents on the railways-a study of injuries to individuals with particular reference to children and to nonfatal trauma. Accid Anal Prev. 10.1016/0001-4575(85)90054-5. 4096788

[22] Schneble CA, Raymond J, Loder RT (2019) The demographics of non-motor vehicle associated railway injuries seen at trauma centers in the United States 2007–2014. Cureus. 10.7759/cureus.5974. PMC6874290 31803556

[23] Rodier SG, DiMaggio CJ, Wall S, et al. (2018) Subway-related trauma: An urban public health issue with a high case-fatality rate. J Emerg Med. 10.1016/j.jemermed.2018.04.015. 29753571

[24] Sousa S, Santos L, Dinis-Oliveira RJ, et al. (2015) Pedestrian fatalities resulting from train-person collisions. Traffic Inj Prev. 10.1080/15389588.2014.914181. 24761944

[25] Akkas M, Ay D, Aksu NM, Gunalp M (2011) 10 year evaluation of train accidents. Turkish J Trauma Emerg Surg. 10.5505/tjtes.2011.66750. 22090331

[26] Chattar-Cora D, Tutela RR, Daum AN, Cromack DT (2007) Experience with railroad injuries at a major urban trauma center serving the United States-Mexico border. J Trauma – Inj Infect Crit Care. 10.1097/TA.0b013e318031cc85 17495711

[27] Rautji R, Dogra TD (2004) Rail traffic accidents: A retrospective study. Med Sci Law. 10.1258/rsmmsl.44.1.67. 14984217

[28] Agalar F, Cakmakci M, Kunt MM (2000) Train-pedestrian accidents. Eur J Emerg Med. 10.1097/00063110-200006000-00008. 11132074

[29] Lin PT, Gill JR (2009) Subway train-related fatalities in New York City: Accident versus suicide. J Forensic Sci. 10.1111/j.1556-4029.2009.01165.x. 19804531

[30] Paniker J, Graham SM, Harrison JW. 2015. Global trauma: The great divide. SICOT-J, 1. 10.1051/SICOTJ/2015019. PMC4849241 27163075

